# Comparison of Different Label-Free Raman Spectroscopy Approaches for the Discrimination of Clinical MRSA and MSSA Isolates

**DOI:** 10.1128/spectrum.00763-22

**Published:** 2022-08-25

**Authors:** Aikaterini Pistiki, Stefan Monecke, Haodong Shen, Oleg Ryabchykov, Thomas W. Bocklitz, Petra Rösch, Ralf Ehricht, Jürgen Popp

**Affiliations:** a Leibniz Institute of Photonic Technology Jena, Jena, Germany; b InfectoGnostics Research Campus Jena, Jena, Germany; c Institute of Physical Chemistry and Abbe Center of Photonics, Friedrich Schiller University, Jena, Germany; d Jena Biophotonics and Imaging Laboratory, Jena, Germany; e Center for Sepsis Control and Care, Jena University Hospital, Jena, Germany; University at Albany, State University of New York

**Keywords:** MRSA, Raman spectroscopy, 785 nm fiber probe, 532-nm single-cell analysis, Raman microscopy, UV resonance Raman spectroscopy, single-cell analysis

## Abstract

Methicillin-resistant Staphylococcus aureus (MRSA) is classified as one of the priority pathogens that threaten human health. Resistance detection with conventional microbiological methods takes several days, forcing physicians to administer empirical antimicrobial treatment that is not always appropriate. A need exists for a rapid, accurate, and cost-effective method that allows targeted antimicrobial therapy in limited time. In this pilot study, we investigate the efficacy of three different label-free Raman spectroscopic approaches to differentiate methicillin-resistant and -susceptible clinical isolates of S. aureus (MSSA). Single-cell analysis using 532 nm excitation was shown to be the most suitable approach since it captures information on the overall biochemical composition of the bacteria, predicting 87.5% of the strains correctly. UV resonance Raman microspectroscopy provided a balanced accuracy of 62.5% and was not sensitive enough in discriminating MRSA from MSSA. Excitation of 785 nm directly on the petri dish provided a balanced accuracy of 87.5%. However, the difference between the strains was derived from the dominant staphyloxanthin bands in the MRSA, a cell component not associated with the presence of methicillin resistance. This is the first step toward the development of label-free Raman spectroscopy for the discrimination of MRSA and MSSA using single-cell analysis with 532 nm excitation.

**IMPORTANCE** Label-free Raman spectra capture the high chemical complexity of bacterial cells. Many different Raman approaches have been developed using different excitation wavelength and cell analysis methods. This study highlights the major importance of selecting the most suitable Raman approach, capable of providing spectral features that can be associated with the cell mechanism under investigation. It is shown that the approach of choice for differentiating MRSA from MSSA should be single-cell analysis with 532 nm excitation since it captures the difference in the overall biochemical composition. These results should be taken into consideration in future studies aiming for the development of label-free Raman spectroscopy as a clinical analytical tool for antimicrobial resistance determination.

## INTRODUCTION

Increasing antimicrobial resistance is a worldwide health issue (1). Staphylococcus aureus is a Gram-positive bacterium that can be found as a colonizer of the skin and mucosal membranes of 20 to 30% of the entire human population (2). It can cause a wide range of localized and metastatic infections that are difficult to treat (3, 4) and is one of the most common causative agents of bacteremia and sepsis (5). Methicillin-resistant Staphylococcus aureus (MRSA) strains are resistant to β-lactams, a broadly used group of antimicrobial agents (6). These strains are classified by the World Health Organization (WHO) as one of the 12 priority pathogens that threaten human health (7). Early detection of S. aureus infection and fast determination of its antibiotic resistance are crucial for the administration of appropriate antibiotic treatment that directly affects disease outcome (8). Delays in its administration are associated with a high risk for mortality (9). However, traditional microbiological techniques, such as MIC determination or disk diffusion test, which have an accuracy that is over 96% (10, 11), require several days to provide information on antimicrobial resistance, forcing physicians to administer empirical treatments that are not always appropriate. This often results in the use of last-line antibiotics and contributes to a further increase of antimicrobial resistance. In order to avoid these delays, molecular diagnostic techniques, such as PCR-based methods, are used for the detection of antimicrobial resistance determinants. These methods also provide <95% accuracy (12), but their high complexity and costs compared to traditional microbiology techniques make them often not suitable for many clinical laboratories (13). Thus, there is an urgent need for an inexpensive, rapid, sensitive, and specific test, with potential for point-of-care use, for the discrimination of MRSA and MSSA that allows the administration of targeted instead of empirical antibiotic treatment without delay.

Raman spectroscopy is one of the promising analytical methods for bacterial cells that have been introduced in the past decades (14). This method detects vibrational modes of the chemical bonds in biomolecules, capturing a unique spectrum for each bacterial target that is often described as an “optical fingerprint” (15). This provides a profile of the overall biochemical composition of the bacteria, even allowing species identification (16, 17), discrimination of multidrug resistance from susceptible isolates (18), as well as epidemiological typing (19–21). In a previous study, it was shown that Raman spectroscopy could differentiate 16 Staphylococcus species with an accuracy of 99% (22).

Many different Raman spectroscopic approaches have been introduced over the years for analyzing biological samples such as cells, bacteria, and tissue (16, 23–27). In bacterial cell analysis, their main differences relate to the application of different excitation wavelengths and whether bulk or single-cell analyses are performed. The different excitation wavelengths provide a different profile of the bacterial biochemical composition, depending on how close the used excitation wavelength is to the absorption spectrum of their biomolecules (28). Concerning cell analysis, single-cell analysis is not suitable for every wavelength due to factors such as photothermal damage (UV) or low signal intensity (near infrared [NIR]). Also, the cell-to-cell heterogeneity deriving from differences in metabolic and cell cycle stages in each bacterial cell is captured in the spectra, leading to high noise production, and could negatively influence the classification accuracy due to misclassifications. In contrast, the low biomass that is required for this type of analysis results in lower incubation times and significantly higher analysis speed (17). In bulk analysis, sample heterogeneity is averaged through the measurement of thousands of cells simultaneously, leading to a better signal-to-noise ratio and an avoidance of fluorescence. However, in order to obtain the large amount of required biomass, a longer incubation time is required.

So far, only very few studies have been published that aim to discriminate MRSA and MSSA strains using label-free Raman spectroscopy. In most of these studies, comparisons were performed between one MRSA and one MSSA strain, using only one excitation wavelength (26, 29, 30), and to our knowledge, no study exists that attempted the differentiation of MRSA and MSSA using UV resonance Raman (UVRR) microspectroscopy.

The aim of the present pilot study was to test and compare three label-free Raman spectroscopic approaches in their efficacy to discriminate MRSA from MSSA strains. For this purpose, three different Raman approaches were applied, namely, 785 nm excitation using a fiber probe directly on the petri dish, UVRR on bulk samples, and single-cell analysis with 532 nm excitation to define the most suitable one for the discrimination of four pairs of MRSA and MSSA strains with highly similar genotypic characteristics.

## RESULTS

**FIG 1:**
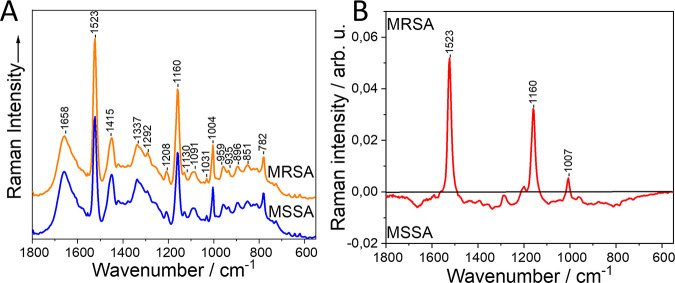
Mean Raman spectrum (A) and difference spectrum (B) of MRSA and MSSA strains by using 785 nm excitation on bacterial colonies directly on the petri dish. arb. u, arbitrary units. Spectra are shifted vertically for clarity.

In Fig. 1, the spectra from the fingerprint area of the bacterial measurements with 785 nm excitation are presented. The most intense, as well as important, peaks are highlighted. In the mean spectrum, Raman signals from main groups of biomolecules can be observed. The exact band assignments of the bands in the mean spectra are shown in Table S3 in the supplemental material, and those of the difference spectra are shown in Table S4. The bands at 1,658, 1,208, 1,031, 1,004, and 851 cm^−1^ can be assigned to proteins, at 1,292, 1,130, 959, and 896 cm^−1^ to lipids, and at 1,091, 953, and 782 cm^−1^ to nucleic acids. Mixed bands of lipids and proteins and proteins and nucleic acids can be seen at 1,337 and 935 cm^−1^, respectively. In the mean spectrum, the characteristic spectral pattern of carotenoids can be observed at 1,523, 1,160, and 1,007 cm^−1^ (29, 31) that represent the most prominent peaks in the difference spectrum. These signals derive mainly from the carotenoid staphyloxanthin, the characteristic golden pigment of S. aureus present in the majority of the strains of this species (32, 33). In the difference spectrum (Fig. 1B), it can be seen that the staphyloxanthin bands are more intense in the MRSA than in the MSSA strains. However, this cannot be generalized since different concentrations of staphyloxanthin are present in different strains independent of their antimicrobial resistance. The differentiation between MRSA and MSSA can be obtained with a balanced accuracy (average of the model’s sensitivity and specificity) of 87.5% (Table 1).

**TABLE 1:** Confusion matrix of classification model for MRSA and MSSA pairs measured with 785 nm excitation directly on the petri dish^*a*^

785 nm excitation	Prediction		Accuracy (%)	Sensitivity (%)	Specificity (%)
	MRSA	MSSA			
MRSA	3	1	87.5	75	100
MSSA	0	4	87.5	100	75

a Correctly identified strains are shown with shading.

**FIG 2:**
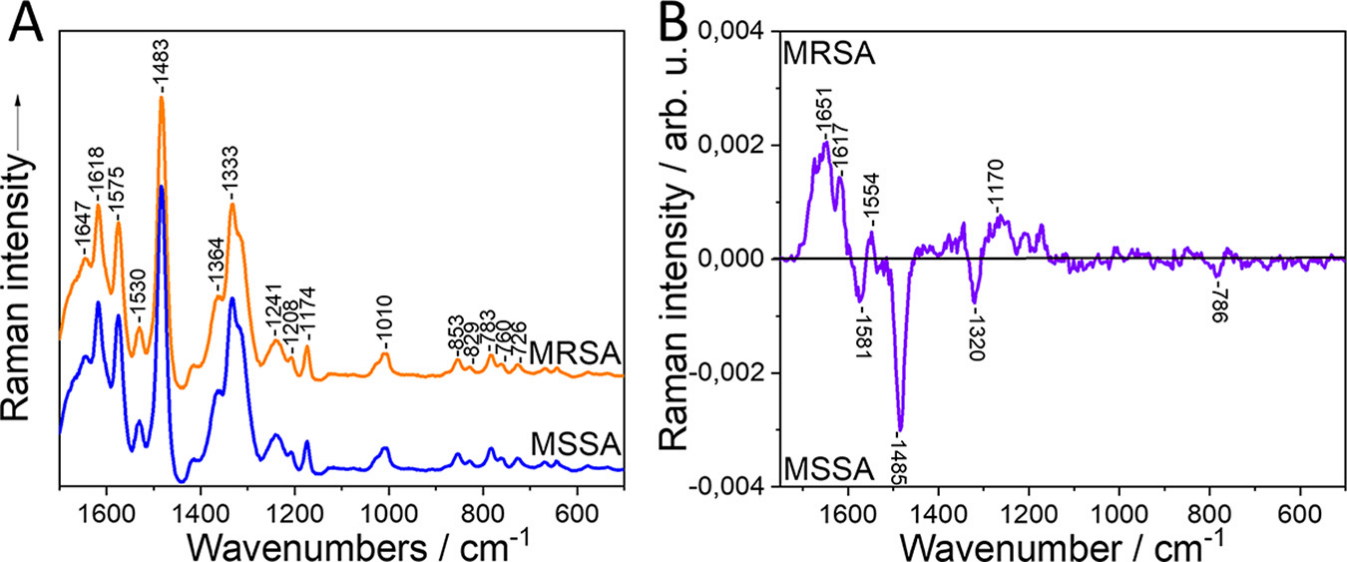
Mean Raman spectrum (A) and difference spectrum (B) of MRSA and MSSA strains by using UVRR. arb. u, arbitrary units. Spectra are shifted vertically for clarity.

UVRR with 244 nm excitation is a promising approach since nucleic acids and aromatic amino acids are in resonance in this excitation. This results in an increase of the Raman signal from these molecules up to 10^6^ orders of magnitude (34, 35), causing their signals to dominate and bands with lower intensity to practically disappear from the spectrum. The antimicrobial resistance is caused by the presence of the resistance gene *mecA* or *mecC* that is located on the mobile genetic element SCC*mec*. It was hypothesized that with UVRR, the resonance effect may reveal these genetic differences, providing a good differentiation between the methicillin-susceptible and -resistant strains. Results are presented in Fig. 2 and Table 2, and the exact band assignments of the mean and difference spectrum are shown in Table S5 and S6, respectively. The mean Raman spectrum consists almost entirely of molecules that are in resonance, with the exception of the amide I band at 1,647 cm^−1^. The peaks at 1,575, 1,530, 1,483, 1,416, 1,364, 1,241, 1,133, 783, 760, and 726 cm^−1^ can be assigned to nucleic acids and at 1,647, 1,618, 1,208, 1,010, 853, and 829 cm^−1^ to proteins. In addition, the bands at 1,174 cm^−1^ originate from superimposed signals from vibrational modes from nucleic acids and protein. In the difference spectrum, it can be seen that in the MRSA strains, the majority of the signals, which were at 1,653, 1,614, and 1,203 cm^−1^, derive from protein, and one combined band of proteins and nucleic acids at 1,170 cm^−1^ is present. In the MSSA, on the other hand, the difference spectrum revealed only nucleic acid peaks, which were at 1,581, 1,536, 1,486, 1,320, and 786 cm^−1^. These results do not support the hypothesis that UVRR can reveal the genetic differences deriving from the presence of the *SCCmec* genetic element. In addition, the balanced accuracy for differentiation is 62.5%, which is very low.

**TABLE 2:** Confusion matrix of classification model for MRSA and MSSA pairs measured with UVRR^*a*^

UVRR detection	Prediction		Accuracy (%)	Sensitivity (%)	Specificity (%)
	MRSA	MSSA			
MRSA	2	2	62.5	50	75
MSSA	1	3	62.5	75	50

a Correctly identified strains are shown with shading.

**FIG 3:**
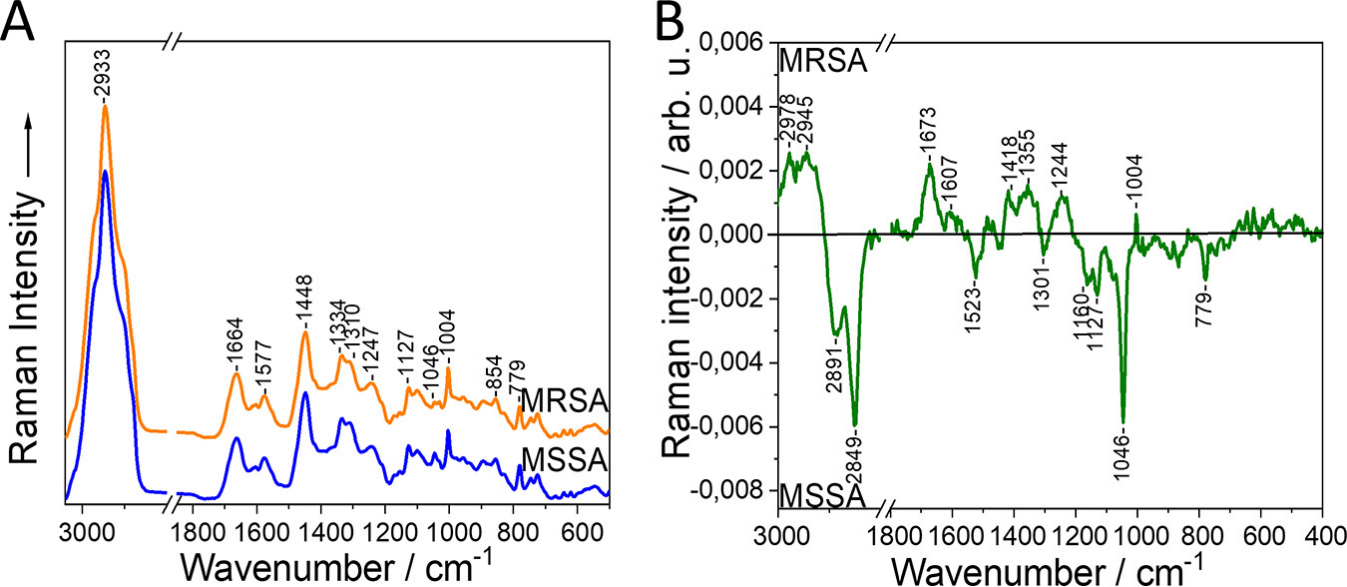
Mean Raman spectrum (A) and difference spectrum (B) of MRSA and MSSA strains by using single-cell analysis with 532 nm excitation. arb. u, arbitrary units. Spectra are shifted vertically for clarity.

A single-cell analysis approach was performed using 532 nm excitation. Results are presented in Fig. 3 and Table 3, and the exact band assignments of the mean and difference spectrum are shown in Table S7 and S8, respectively. In the mean Raman spectrum, it can be observed that all major biomolecule groups (proteins, lipids, and nucleic acids) present in a bacterial cell are represented in the Raman bands of both the MRSA and the MSSA strains. These results agree with the S. aureus metabolome as published previously, where all these biomolecule groups were represented (36). In the mean Raman spectrum, bands deriving from lipid can be seen at 2,933, 2,888, and 1,127 cm^−1^, from proteins at 1,664, 1,310, 1,247, 1,046, 1,004, and 854 cm^−1^, and from nucleic acid at 1,577, 779, and 725 cm^−1^. Mixed signals from lipids and proteins and proteins and nucleic acids are present at 1,448 and 1,334 cm^−1^, respectively. In the difference spectrum, signals from all biomolecules are spread between the MRSA and MSSA strains, with no particular pattern. The MRSA shows lipid peaks at 2,978 and 2,945 cm^−1^ and the MSSA at 2,891, 2,849, 1,301, 1,160, and 1,127 cm^−1^. The protein bands in the MRSA are revealed at 1,673, 1,607, 1,244, and 1,004 cm^−1^ and in the MSSA at 1,046 cm^−1^. The nucleic acids appear at 1,487 cm^−1^ in the MRSA and 1,523 and 779 cm^−1^ in the MSSA strains. Also, two mixed bands of proteins, nucleic acids, and lipids and of proteins and nucleic acids are present in the MRSA strains at 1,418 and 1,355 cm^−1^, respectively. The peaks at 1,004, 1,160 and 1,523 cm^−1^ were not assigned to the carotenoid staphyloxanthin because it has been previously shown that photodecomposition occurs for this biomolecule when exposed to 532 nm laser excitation (37, 38), and, in addition, the bands do not show the characteristic spectral pattern of carotenoids that was shown in the 785-nm excitation. It can be observed that all major biomolecule groups were represented in both the MRSA and the MSSA and that they appeared with no particular pattern in the distribution of the biomolecules, showing the overall biochemical profile of the bacterial strains.

**TABLE 3:** Confusion matrix of classification model for MRSA and MSSA pairs measured with 532 nm excitation single-cell analysis^*a*^

532 nm excitation	Prediction		Accuracy (%)	Sensitivity (%)	Specificity (%)
	MRSA	MSSA			
MRSA	3	1	87.5	75	100
MSSA	0	4		100	75

a Correctly identified strains are shown with shading.

## DISCUSSION

In the present study, it is shown that the Raman approach of choice for the discrimination of MRSA and MSSA isolates should be single-cell analysis using 532 nm excitation. An accuracy for differentiation of 87.5% could be achieved, and biochemical interpretation of the spectra showed that this approach provides an overall biochemical profile of the bacterial cells. UVRR yielded an accuracy of 62.5%, which is very low. The accuracy of 87.5% that was provided by the Raman analysis with 785 nm excitation directly on the bacterial colonies derived from a virulence factor that has no relation to methicillin resistance.

The discrimination of MRSA and MSSA when using 785 nm excitation directly on the bacterial colonies is based on the prominent staphyloxanthin bands that show higher intensity in the MRSA strains. Staphyloxanthin has been associated with S. aureus virulence since it acts as a shield against neutrophil oxidative burst, a host response mechanism to infections (39, 40), and has no relation to antimicrobial resistance mechanisms. In a previous study aiming to discriminate MRSA and MSSA using 785 nm excitation directly on bacterial colonies, similar results were obtained (29). There, it was found that pigmented and nonpigmented S. aureus mutants could be distinguished based on analysis of three spectral regions (765 to 934, 1,431 to 1,464, and 1,495 to 1,544 cm^−1^), one of which can be assigned to staphyloxanthin. Furthermore, when one MRSA and its mutants were compared to one MSSA isolate, a decrease in pigmentation in the MSSA was evident that was shown by decreased ratios of 1,523/1,004- and 1,456/1,004-cm^−1^ intensities. However, it has to be mentioned that the authors of this study (29) did not consider the contribution of the C-CH_3_ in-plane rocking vibrations of carotenoids in the phenylalanine band at 1,004 cm^−1^, and thus, the comparisons performed in both used ratios included carotenoid band contributions. This finding is similar to our finding of increased carotenoid expression in MRSA isolates. However, discrimination based on carotenoid signals cannot be considered relevant since they have no impact on the mechanism of methicillin resistance whatsoever.

UVRR microspectroscopy was considered a very promising approach since it was expected that the presence of the resistance genes in the MRSA strain would result in differences in their spectra from MSSA. However, despite the resonant enhancement of signal from nucleic acid, UVRR was not sensitive enough to capture these very small differences in the size of the strain’s genome caused by the SCC*mec* genetic element. The genome of S. aureus has an average size of ~2.8 Mbp (41) and varies within a range of ±0.3 Mbp. The size of the SCC*mec* genetic element ranges between 21 and 67 kbp (42), comprising only a very small subset of the whole genome. In addition, the variable presence of numerous mobile genetic elements in S. aureus contributes to large strain-to-strain variations in genome size that could also be a source of misclassifications due to the contribution of other genes, or mobile genetic elements, in the Raman spectrum of the strains. Thus, UVRR cannot be considered the approach of choice for the discrimination of MRSA from MSSA.

Single-cell analysis with 532 nm excitation provided high accuracy for differentiation, and the biochemical interpretation of the Raman bands showed an overall biochemical profiling of the bacterial cells. Metabolomics studies have shown that the presence of antimicrobial resistance genes alters the metabolic profile of bacteria. When comparing MRSA and MSSA strains, the metabolic differences were associated mainly with the capsular and cell wall biosynthesis pathways, the accumulation of bacillithiol, and the purine/pyrimidine metabolism (36). Similar findings were obtained when comparing the metabolomes of 6 isogenic clinical S. aureus strains that were susceptible (Dap-S) and nonsusceptible (Dap-NS) to daptomycin (43). Growth delays during the transition between exponential and postexponential phase were found in Dap-NS that were combined with lower fitness, decrease of carbon flow into the tricarboxylic acid (TCA) cycle accompanied by increase into pathways associated with wall teichoic acid and peptidoglycan biosynthesis, increase in purine/pyrimidine biosynthesis, and alterations in oxidative phosphorylation (43). Such biochemical alterations might be captured in the Raman spectra of the present study, allowing the discrimination of the MRSA and MSSA with a balanced accuracy of 87.5%. However, it is not possible to define the exact biochemical changes using Raman spectroscopy since this method mainly provides information about the major biomolecule groups without being, most of the time, capable of defining the exact biomolecules involved. Exceptions are, for example, carotenoids that are seen in the 785-nm excitation. In the UVRR and 532-nm excitation, spectra bands from specific nucleotides and aromatic amino acids bands as cytosine and tyrosine can be seen; however, it is challenging to associate these signals with specific genes expressed or specific proteins present in the cell. In addition, the presence of overlapping signals from different molecules within the spectra and the absence of a unique database for Raman analysis of bacteria increase, at this point, the difficulty for biological interpretation of the Raman spectra. To investigate the cell mechanism that explains where the captured differences between MRSA and MSSA strains derive from, the application of complex metabolome analysis is required, but this is out of the scope of this pilot study.

The main disadvantage of single-cell analysis is the high in-sample heterogeneity due to cell-to-cell differences in growth stage and metabolism (44). In a previous study, single-cell analysis with 532 nm excitation was applied on 3 MRSA and 2 MSSA strains in the presence and absence of cefoxitin (30). This exposure causes minimal changes in the resistant bacteria but major changes in the sensitive bacteria that can be detected compared to the unchallenged control. It was observed that when challenged with the antibiotic, changes occur in the MSSA strains that allow a visual separation from the unchallenged bacteria. However, classification showed a high variance in prediction, ranging from about 33 to 100%, that is caused by the high in-sample variability of the measured single cells (30). In studies using Gram-negative isolates (45–47) and Enterococcus faecalis (48), it has been shown that Raman spectroscopy can be used as an accurate susceptibility test when the isolates are previously exposed to antibiotics. In the present study, a high accuracy for differentiation could be yielded without previous antibiotic challenge, showing that susceptibility testing using Raman microspectroscopy can be further simplified, providing accurate results even more easily and quickly. The Raman spectra acquisition time is ~30 min/sample for the 532-nm excitation, and when including the sample preparation, drying, and data analysis steps, it goes up to ~2 h. This is similar to PCR when considering the sample preparation steps and is significantly lower than the 24 h required for automatic antimicrobial susceptibility testing (AST) devices. However, within the Raman workflow, similar to PCR and automatic AST, the plating step cannot be avoided at this point, as well as the requirement for Raman device and trained personnel. But despite the advantage in time compared to automatic AST testing, the lack of expensive consumables of this label-free method also provides a significant advantage over both automatic AST and PCR. The low bacterial biomass required for single-cell analysis could enable culture free-approaches (16, 49, 50) for bacterial isolation that can further provide a time benefit of 1 day by avoiding the initial plating step of the patient sample. Nevertheless, these approaches are highly sample specific and need to be further investigated and standardized before considering their application in clinical laboratory practice.

The findings of a very recent study using single-cell analysis with 532 and 785 nm excitation were indicative that a discrimination of MRSA and MSSA isolates could be achieved using Raman spectroscopy (51). However, in the present study, a systematic investigation toward this direction is performed using larger numbers of highly similar isolates, analyzing biological replicates, and investigating different Raman spectroscopy approaches, aiming to reveal both the potential of this technique in discriminating MRSA and MSSA as well as the technical details that lead to the best results.

It has to be mentioned that S. aureus is a very complex species containing an extremely variable accessory genome that is often strain specific and can carry a large variety of genes (52). All of these factors increase the difficulty of discriminating methicillin-resistant and -susceptible strains in a label-free way, and the need exists for larger studies to be performed in the future. However, the present study has demonstrated that the best approach for this attempt is by applying single-cell analysis with 532 nm excitation. Issues such as the low accuracy for differentiation compared to the standard methods need to be addressed in future studies, and as a next step, fine-tuning needs to be performed in order to make this method applicable in clinical settings.

In the present study, it is shown that label-free Raman microspectroscopy with 532 nm excitation and single-cell analysis is the most suitable approach for the discrimination of MRSA and MSSA strains, providing a balanced accuracy of 87.5%. This method captures the overall biochemical composition of the bacterial cells and might detect the differences in cell metabolism that are present in MRSA and MSSA strains.

Despite that UVRR was considered to be a very promising approach, it showed to be not sensitive enough to capture the fine differences that discriminate MRSA and MSSA strains. Bulk analysis of bacterial colonies directly on the petri dish using a fiber probe and 785 nm excitation was not suitable since, in this wavelength, the differences derived from the dominant staphyloxanthin signals in the MRSA that is not associated with antimicrobial resistance. The findings of the present study can guide future studies that aim to discriminate MRSA in larger cohorts and setups. Classification models can be built, capable of providing good classification of unknown strains, making this method suitable for application in clinical laboratory settings. The development of Raman spectroscopy as a novel, rapid, label-free diagnostic tool for MRSA discrimination could allow for overcoming the limitations of the current highly cost- and time-consuming methods used in clinical diagnostics, improving patient care and limiting unnecessary use of last-line antibiotics.

## MATERIALS AND METHODS

### Bacterial strains.

In order to investigate the ability of Raman spectroscopy to discriminate MRSA and MSSA isolates, we aimed to keep the differences between the strains as limited as possible. For this purpose, four pairs of MRSA and MSSA were selected from our biobase. In order to be as close as possible to real conditions, clinical isolates were selected. These are strains that have developed in the clinical environment and are the type of strains that are isolated in microbiology laboratories for resistance determination. Two pairs belonged to the clonal complex CC8 and two to the clonal complex CC361. Each of the clonal complexes included an isogenic pair and one pair of independent strains that were highly similar to the isogenic pairs. The isogenic pairs consisted of an MRSA strain and an *mecA*-negative derivative that lost its SCC*mec* element during repeated *in vitro* passages. Additional isolates were selected based on their array hybridization profiles (see below) to be as similar to the respective isogenic pair as possible. For the characterization of the isolates, microarray analysis was performed using StaphyType DNA microarrays (Alere Technologies GmbH, Jena, Germany). We analyzed 333 different targets, corresponding to 170 distinct genes and their allelic variances, including species-specific genes, typing markers, and toxin and resistance genes. Details on the gene products, alleles, probes, and primers of this assay have been published elsewhere (53, 54). Microarray analysis results of the used strains are shown in Table S1 in the supplemental material. Concerning the resistance profiles of the strains, all strains considered MRSA were positive for the *mecA* gene providing methicillin resistance. In addition, all isolates carry the gene *fosB*, providing resistance to fosfomycin through the protein FosB, and the chromosomal efflux pump gene *sdrM*, providing resistance to the fluoroquinolone norfloxacin. The isogenic pair AUSTR-07-16859 carries the erythromycin ribosome methylenation gene *ermC*, and in the MRSA strain of this pair, the widely distributed qac efflux pump gene *qacC* and *qacC*(SA) are present. This is most likely due to the presence of these genes on a plasmid that was lost during transformation. The strain 08V15773 also carries the efflux pump gene *qacA*, and strain UAE-Abu Dhabi-020 carries the gene *aaCA-aphD*, providing resistance to aminoglycosides.

### Sample preparation and Raman spectroscopy measurements.

Bacteria were plated from −80°C storage onto nutrient agar (NA) plates (Carl Roth, Karlsruhe, Germany) and were incubated for 24 h at 37°C. This was performed on every measurement day for all isolates as part of the sample preparation.

### Analysis with 785 nm excitation.

To minimize the influence of the dishes’ Raman signal on the bacterial spectra, 2 to 3 bacterial colonies were transferred from the agar plate onto petri dishes made of stainless steel (Bochem, Weilburg, Germany) with a diameter of 75 mm and containing 8 mL neuraminidase (NA), and they were incubated for 16 to 18 h at 37°C. Bulk Raman measurements were afterward performed on bacterial colonies directly on that petri dish by using the fiber probe. Raman spectra were obtained with a Raman system (Kaiser Optical Systems, Ann Arbor, MI, USA) coupled with a 785-nm single-mode diode laser (Toptica, Gräfelfing, Germany) as previously described (55). The sample was focused through a Raman fiber probe (InPhotonics, Norwood, MA, USA) with a focal spot diameter of ~50 μm and depth of field of ~200 μm, delivering ~200 mW power to the sample plane. The corresponding irradiance was ~10^4^ W/cm^2^. After passing a holographic transmissive grating, the scattered Raman signal was detected on a thermoelectrically cooled, back-illuminated, open-electrode charge-coupled-device (CCD) chip (Andor, Northern Ireland), providing a spectral resolution of ~4 cm^−1^. Each Raman spectrum was obtained from a single microbial colony with 10 s integration time and three accumulations. For each strain, three biological replications (batches) were measured on three consecutive days, consisting of ~20 spectra/batch. Results are shown in Fig. 1.

### UVRR.

Two to three loopfuls of biomass were transferred from the agar plate into 20 mL nutrient broth (NB) (Carl Roth) and were incubated for 1 h in a shaking incubator at 37°C and 120 rpm. After the incubation, some strains were at the end of the lag phase, and some were at the beginning of the exponential phase. Growth curves are shown in Fig. S1. We transferred 1.5 mL of the inoculum into three separate Eppendorf tubes, and they were heat inactivated at 99°C for 10 min followed by 3 consecutive washings with 1 mL deionized water (DI) using centrifugation at 5,000 × *g* for 5 min (Rotina 380R; Hettich) (18, 56). The bacterial pellet was then resuspended in 30 μL DI, and all three replicates were placed separately onto a fused-silica slide (B&M Optik GmbH, Germany) to air-dry at room temperature for ~1 h. To verify the heat inactivation, bacteria were plated onto NA plates and incubation for 24 h at 37°C, and no growth could be detected.

Raman spectra were collected using a Raman setup (HR800; Horiba Jobin-Yvon) coupled with an argon-ion laser (Coherent Innova 300; FReD) with a focal length of 800 mm. We produced 244 nm by doubling the frequency of the 488-nm line. Maximal laser power of 20 mW was used, leading to about 0.5 mW on the sample. The laser was directed and focused on the sample through a 40× antireflection-coated objective (LMU; numerical aperture, 0.5; UVB). Backscattered Raman light was collected through a 400-μm entrance slit into a 2,400-line/mm grating and detected by a nitrogen-cooled CCD camera, leading to a spectral resolution of 2 cm^−1^. In order to avoid burning the sample, the sample stage was constantly rotated in a spiral manner during measurement. Each measurement consisted of 10 consecutive measurements of 15-s integration that were afterward averaged to reduce noise. Three biological replicates (batches) were measured for each strain on different days. Strain pairs AUSTR07-16859 and AUSTR05-15441 were measured on 3 consecutive days. The other two strain pairs were measured in the same manner 1.5 months later. Each replicate consisting of 25 measurements was collected from 3 fused silica slides to avoid remeasuring burned areas of the sample. A total of 600 spectra per isolate were collected. Results are shown in Fig. 2.

### Analysis with 532 nm excitation.

A loopful of biomass was transferred from the agar plate into 5 mL NB and incubated overnight in a shaking incubator at 37°C and 120 rpm. One milliliter of the bacterial culture was washed 3 consecutive times with DI as described above and reconstituted into 1 mL DI. Ten microliters of the bacterial cell suspension were placed in ~1-μL droplets onto an Ni-foil disc and left to air-dry at room temperature for ~30 min.

Individual bacterial cells were measured using a Bio Particle Explorer (Microbio ID, 0.5; RapID) Raman microscope coupled with a 532-nm frequency-doubled solid-state Nd:YAG diode pumped laser (LCM-S-111; Laser-Export Company Ltd.). The laser beam was focused on the sample through a ×100 magnification objective (MPLFLN 100×; numerical aperture, 0.9; Olympus Corporation). Maximum laser power was approximately 16 mW, leading to ~3.5 mW on the bacterial cell. Backscattered Raman light was focused to a single-stage monochromator (HE 532; Horiba Jobin-Yvon) equipped with a 920-line/mm grating and collected with a thermoelectrically cooled CCD camera (DV401A-BV; Andor Technology), leading to spectral resolution of ~10 cm^−1^. For each bacterial cell, two consecutive Raman spectra were measured at the same position, which were afterward combined using an integration time of 15 s. On every measurement day, 60 single-cell spectra per strain were collected. For each isolate, three biological replicates (batches) were measured on 3 consecutive days. A total of 180 spectra per isolate were collected. Results are shown in Fig. 3.

### Statistical analysis.

Preprocessing and data analysis were done using the RAMANMETRIX software (https://ramanmetrix.eu) (57). The number of total spectra, the spectra per batch, and spectra per strain measured, as well as the total number of spectra analyzed, are shown in Table S9. Prior to analysis, several preprocessing steps were performed, including despiking with a manual threshold as described before by Ryabchykov et al. (58) and wavenumber calibrated with polynomial fit function with a degree of 3 for the 532 and 785 nm excitation and 2 for the UVRR spectra. Standard reference spectra of 4-acetamidophenol were used for wavenumber calibration of 532 and 785 nm excitation, and standard reference spectra of polystyrene were used for wavenumber calibration in the UVRR. Standard reference spectra were measured on each measurement day prior to the samples. Afterward, sample spectra were baseline corrected using a sensitive nonlinear iterative peak (SNIP) clipping algorithm with 40 iterations and vector normalization. Spectra were then truncated to the relevant range of 500 to 1,900 cm^−1^ for UVRR, 400 to 3,050 cm^−1^ (excluding the silent region at 1,800 to 2,800 cm^−1^) for the 532-nm excitation data, and 500 to 1,800 cm^−1^ for the 785-nm excitation (59). In addition, for the 532-nm excitation, burned spectra were removed automatically from the data set using an in-house R script (60), and the data were filtered by a 0.9 correlation filter to remove any remaining outliers, with 88.6% of spectra passing the filter.

For each excitation wavelength classification, models were calculated in order to differentiate MRSA from MSSA isolates. A principal-component analysis combined with support vector machine (PCA-SVM) approach was used for all models, and the number of principal components used was optimized automatically based on the results of a leave-one-batch-out cross-validation (LOBOCV) as described by Guo et al. (61). With this validation method, a model is calculated, repeatedly excluding one batch that is then predicted by the constructed model. For the UVRR data, the PCA-SVM model was calculated, based on 4 principal components (PCs), the data for the 532-nm excitation were based on 7 PCs, and data for the 785-nm excitation were based on 31 PCs. Results from the classification models are shown in Table S10, and results of the percentage of correctly identified spectra per strain are shown in Table S11. After the calculation of the classification models, a majority vote was taken to classify each strain individually. The purpose of this was to suppress in-sample heterogeneity that sometimes leads to the incorrect classification of spectra. Results from majority voting per batch and strain are provided in Table S2, showing the batch-to-batch heterogeneity. Balanced accuracy was calculated by averaging the model’s sensitivity and specificity. Finally, spectra were visualized using OriginPro, version 2018b (OriginLab Corporation, Northampton, USA).

### Data availability.

Data, metadata, and a data description file are available on Zenodo at https://zenodo.org/record/6603209#.YvQ8X3bMK3A.
