# Supplementary material for: Comparison of Different Label-Free Raman Spectroscopy Approaches for the Discrimination of Clinical MRSA and MSSA Isolates
Source: Microbiol Spectr. 2022 Aug 25;10(5):e00763-22. doi: 10.1128/spectrum.00763-22 (PMC9603629; doi:10.1128/spectrum.00763-22)
Supplement: Supplemental file 1 — Supplemental material. Download spectrum.00763-22-s0001.pdf, PDF file, 0.7 MB [file spectrum.00763-22-s0001.pdf]

Comparison of different label-free Raman spectroscopy approaches for the discrimination of clinical MRSA and MSSA isolates

Aikaterini Pistiki[a], Stefan Monecke [a,b,f], Haodong Shen[a,c], Oleg Ryabchykov[a,c], Thomas W. Bocklitz[a,c], Petra Rösch[a,c], Ralf Ehricht[a, b, c] Jürgen Popp[a,b,c,d,e #]

[a]Leibniz Institute of Photonic Technology Jena, Albert-Einstein-Str. 9, 07745 Jena, Germany

[b]InfectoGnostics Research Campus Jena, Philosophenweg 7, 07743 Jena, Germany

[c]Institute of Physical Chemistry and Abbe Center of Photonics, Friedrich Schiller University, Lessingstraße 10, 07743 Jena, Germany

[d]Jena Biophotonics and Imaging Laboratory, Albert-Einstein-Str. 9, 07745 Jena, Germany

[e]Center for Sepsis Control and Care, Jena University Hospital, Am Klinikum 1, 07747 Jena, Germany

Running Head: Label-free Raman approaches for MRSA and MSSA discrimination

#Address correspondence to Jürgen Popp juergen.popp@uni-jena.de,

Table S1: Microarray analysis of the used strains containing 170 distinct genes and their allelic variances. Abbreviations: NEG: negative, POS: positive, AMP: Ambiguous NA: Not available.

		MRSA	MSSA	MRSA	MSSA	MRSA	MSSA	MRSA	MSSA
		AUSTR-07-16859	AUSTR-07-16859	08V15773	MRSA2010-174	AUSTR-05-15441	AUSTR-05-15441	UAE-Abu Dhabi-020	UAE-Dubai-80-MS 1368.9/09
SPECIES MARKER	Ribos. STAU	POS	POS	POS	POS	POS	POS	POS	POS
	gapA	POS	POS	POS	POS	POS	POS	POS	POS
	katA	POS	POS	POS	POS	POS	POS	POS	POS
	CoA	POS	POS	POS	POS	POS	POS	POS	POS
	nuc1	POS	POS	POS	POS	POS	POS	POS	POS
STAPHYLOXANTHIN BIOSYNTHESIS OPERON	crtM-nonST93	N/A	POS	POS	N/A	POS	POS	POS	N/A
	crtM-ST93	N/A	NEG	NEG	N/A	NEG	NEG	NEG	N/A
	crtN	N/A	POS	POS	N/A	POS	POS	POS	N/A
	crtO	N/A	POS	POS	N/A	POS	POS	POS	N/A
	crtP	N/A	POS	POS	N/A	POS	POS	POS	N/A
REGULATORY GENES	sarA	POS	POS	POS	POS	POS	POS	POS	POS
	saeS	POS	POS	POS	POS	POS	POS	POS	POS
	vraS	POS	POS	POS	POS	POS	POS	POS	POS
	agrI (total)	POS	POS	POS	POS	POS	POS	POS	POS
	agrII (total)	NEG	NEG	NEG	NEG	NEG	NEG	NEG	NEG
	agrIII (total)	NEG	NEG	NEG	NEG	NEG	NEG	NEG	NEG
	agrIV (total)	AMB	NEG	AMB	NEG	NEG	NEG	NEG	AMB
	hld	POS	POS	POS	POS	POS	POS	POS	POS
METHICILLIN RESISTANCE AND SCCmec TYPING	mecA	POS	NEG	POS	NEG	POS	NEG	POS	NEG
	delta_mecR	POS	AMB	POS	NEG	POS	NEG	NEG	NEG
	ugpQ	POS	NEG	POS	NEG	POS	NEG	POS	NEG
	ccrA-1	NEG	NEG	NEG	NEG	NEG	NEG	NEG	NEG
	ccrB-1	NEG	NEG	NEG	NEG	NEG	NEG	NEG	NEG
	plsSCC (COL)	NEG	NEG	NEG	NEG	NEG	NEG	NEG	NEG
	Q9XB68-dcs	POS	NEG	POS	NEG	POS	NEG	NEG	NEG
	ccrA-2	POS	NEG	POS	NEG	POS	NEG	NEG	NEG
	ccrB-2	POS	NEG	POS	NEG	POS	NEG	NEG	NEG
	kdpA-SCC	NEG	NEG	NEG	NEG	NEG	NEG	NEG	NEG
	kdpB-SCC	NEG	NEG	NEG	NEG	NEG	NEG	NEG	NEG
	kdpC-SCC	NEG	NEG	NEG	NEG	NEG	NEG	NEG	NEG
	kdpD-SCC	NEG	NEG	NEG	NEG	NEG	NEG	NEG	NEG
	kdpE-SCC	NEG	NEG	NEG	NEG	NEG	NEG	NEG	NEG
	mecI	NEG	NEG	NEG	NEG	NEG	NEG	NEG	NEG
	mecR	NEG	NEG	NEG	NEG	NEG	NEG	NEG	NEG
	xylR	NEG	NEG	NEG	NEG	NEG	NEG	NEG	NEG
	ccrA-3	NEG	NEG	NEG	NEG	NEG	NEG	NEG	NEG
	ccrB-3	NEG	AMB	POS	NEG	NEG	NEG	NEG	NEG
	merA	NEG	NEG	NEG	NEG	NEG	NEG	NEG	NEG
	merB	NEG	NEG	NEG	NEG	NEG	NEG	NEG	NEG
	ccrAA (MRSAZH47)_probe 1	NEG	NEG	NEG	NEG	NEG	NEG	POS	NEG
	ccrAA (MRSAZH47)_probe 2	NEG	NEG	NEG	NEG	NEG	NEG	POS	NEG
	ccrC (85-2082)	NEG	NEG	NEG	NEG	NEG	NEG	POS	NEG
	ccrA-4	NEG	NEG	NEG	NEG	NEG	NEG	NEG	NEG
	ccrB-4	NEG	NEG	NEG	NEG	NEG	NEG	NEG	NEG
	mecC	N/A	NEG	NEG	N/A	NEG	NEG	NEG	N/A
	blaZ-SCCmec XI	N/A	NEG	NEG	N/A	NEG	NEG	NEG	N/A
RESISTANCE : PENICILLINASE	blaZ	POS	POS	POS	POS	POS	POS	POS	POS
	blaI	POS	POS	POS	POS	POS	POS	POS	POS
	blaR	POS	POS	POS	POS	POS	POS	POS	POS
RESISTANCE : MLS-ANTIBIOTICS	erm(A)	NEG	NEG	NEG	NEG	NEG	NEG	NEG	NEG
	erm(B)	N/A	N/A	NEG	NEG	NEG	NEG	NEG	NEG
	erm(C)	POS	POS	NEG	NEG	NEG	NEG	NEG	NEG
	linA (lnu(A))	NEG	NEG	NEG	NEG	NEG	NEG	NEG	NEG
	msrA	NEG	NEG	NEG	NEG	NEG	NEG	NEG	NEG
	mefA	NEG	NEG	NEG	NEG	NEG	NEG	NEG	NEG
	mpbBM (mpB(C))	NEG	NEG	NEG	NEG	NEG	NEG	NEG	NEG
	vatA	NEG	NEG	NEG	NEG	NEG	NEG	NEG	NEG
	vatB	NEG	NEG	NEG	NEG	NEG	NEG	NEG	NEG
	vga(A)	NEG	NEG	NEG	NEG	NEG	NEG	NEG	NEG
	vga(A) (BM 3327)	NEG	NEG	NEG	NEG	NEG	NEG	NEG	NEG
RESISTANCE : AMINOGLYSOSIDES	vgb	NEG	NEG	NEG	NEG	NEG	NEG	NEG	NEG
	aacA-aphD	NEG	NEG	NEG	NEG	NEG	NEG	POS	NEG

	aadD	NEG	NEG	NEG	NEG	NEG	NEG	NEG	NEG
	aphA3	POS	POS	NEG	NEG	NEG	NEG	POS	POS
RESISTANCE : MISCELLANEOUS GENES	sat	POS	POS	NEG	NEG	NEG	NEG	POS	POS
	dfrA	NEG	NEG	NEG	NEG	NEG	NEG	NEG	NEG
	far1	NEG	NEG	NEG	NEG	NEG	NEG	NEG	NEG
	Q6GD50 (fusC)	NEG	NEG	NEG	NEG	NEG	NEG	NEG	NEG
	mupR	NEG	NEG	NEG	NEG	NEG	NEG	NEG	NEG
	tet(K)	NEG	NEG	NEG	NEG	NEG	NEG	POS	POS
	tet(M)	NEG	NEG	NEG	NEG	NEG	NEG	NEG	NEG
	cat	NEG	NEG	NEG	NEG	NEG	NEG	NEG	NEG
	cat (pC221)	NEG	NEG	NEG	NEG	NEG	NEG	NEG	NEG
	cat (pc223)	NEG	NEG	NEG	NEG	NEG	NEG	NEG	NEG
	cat (pMC524)	NEG	NEG	NEG	NEG	NEG	NEG	NEG	NEG
	cat (pSBK203R)	NEG	NEG	NEG	NEG	NEG	NEG	NEG	NEG
	cfr	NEG	NEG	NEG	NEG	NEG	NEG	NEG	NEG
	fexA	NEG	NEG	NEG	NEG	NEG	NEG	NEG	NEG
	fosB	POS	POS	POS	POS	POS	POS	POS	POS
	fosB-plasmid	NEG	NEG	NEG	NEG	NEG	NEG	NEG	POS
RESISTANCE : EFFLUX SYSTEMS	qacA	NEG	NEG	POS	NEG	NEG	NEG	NEG	NEG
	qacC	POS	NEG	NEG	NEG	NEG	NEG	NEG	NEG
	qacC (cons)	NEG	NEG	NEG	NEG	NEG	NEG	NEG	NEG
	qacC (equine)	NEG	NEG	NEG	NEG	NEG	NEG	NEG	NEG
	qacC (SA5)	POS	NEG	NEG	NEG	NEG	NEG	NEG	NEG
	qacC (Ssap)	NEG	NEG	NEG	NEG	NEG	NEG	NEG	NEG
	qacC (ST94)	NEG	NEG	NEG	NEG	NEG	NEG	NEG	NEG
	sdrM	POS	POS	POS	POS	POS	POS	POS	POS
RESISTANCE : GLYCOPEPTIDES	vanA	NEG	NEG	NEG	NEG	NEG	NEG	NEG	NEG
	vanB	NEG	NEG	NEG	NEG	NEG	NEG	NEG	NEG
	vanZ	NEG	NEG	NEG	NEG	NEG	NEG	NEG	NEG
VIRULENCE : TOX.SCHOCK.TOXIN	tst1 (consensus)	NEG	NEG	NEG	NEG	POS	POS	NEG	NEG
	tst1 ("human" allele)	NEG	NEG	NEG	NEG	POS	POS	NEG	NEG
	tst1 ("bovine" allele)	NEG	NEG	NEG	NEG	NEG	NEG	NEG	NEG
VIRULENCE : ENTEROTOXINS	entA	NEG	NEG	NEG	NEG	NEG	NEG	NEG	NEG
	entA (320E)	NEG	NEG	NEG	NEG	NEG	NEG	NEG	NEG
	entA (N315) / entP	NEG	NEG	NEG	NEG	NEG	NEG	NEG	NEG
	entB	NEG	NEG	NEG	NEG	POS	POS	NEG	NEG
	entC	NEG	NEG	NEG	NEG	NEG	NEG	NEG	NEG
	entD	NEG	NEG	NEG	POS	NEG	NEG	NEG	NEG
	entE	NEG	NEG	NEG	NEG	NEG	NEG	NEG	NEG
	entG	NEG	NEG	NEG	NEG	POS	POS	POS	POS
	entH	NEG	NEG	NEG	NEG	NEG	NEG	NEG	NEG
	entI	NEG	NEG	NEG	NEG	POS	POS	POS	POS
	entJ	NEG	NEG	NEG	POS	NEG	NEG	NEG	NEG
	entK	POS	POS	POS	POS	NEG	NEG	NEG	NEG
	entL	NEG	NEG	NEG	NEG	NEG	NEG	NEG	NEG
	entM	NEG	NEG	NEG	NEG	POS	POS	POS	POS
	entN (cons)	NEG	NEG	NEG	NEG	POS	POS	POS	POS
	entN (other than RF122)	NEG	NEG	NEG	NEG	POS	POS	POS	POS
	entO	NEG	NEG	NEG	NEG	POS	POS	POS	POS
	egc (total)	NEG	NEG	NEG	NEG	POS	POS	POS	POS
	entQ	POS	POS	POS	POS	NEG	NEG	NEG	NEG
	entR	NEG	NEG	NEG	POS	NEG	NEG	NEG	NEG
	entU	NEG	NEG	NEG	NEG	POS	POS	POS	POS
	entCM14 probe1	NEG	NEG	NEG	NEG	NEG	NEG	NEG	NEG
	entCM14 probe2	NEG	NEG	NEG	NEG	NEG	NEG	NEG	NEG
VIRULENCE : HLG AND LEUKOCIDINS	lukF	POS	POS	POS	POS	POS	POS	POS	POS
	lukS	POS	POS	POS	POS	POS	POS	POS	POS
	lukS (ST22+ST45)	POS	POS	NEG	AMB	AMB	AMB	AMB	AMB
	hlgA	POS	AMB	POS	POS	POS	POS	POS	POS
	lukF-PV	POS	POS	POS	POS	NEG	NEG	NEG	NEG
	lukS-PV	POS	POS	POS	POS	NEG	NEG	NEG	NEG
	lukF-PV (P83)	NEG	NEG	NEG	NEG	NEG	NEG	NEG	NEG
	lukM	NEG	NEG	NEG	NEG	NEG	NEG	NEG	NEG
	lukD	POS	POS	POS	POS	POS	POS	POS	POS
	lukE	POS	POS	POS	POS	POS	POS	POS	POS
	lukX	POS	NEG	POS	POS	POS	POS	POS	POS
	lukY	POS	POS	POS	POS	POS	POS	POS	POS
	lukY (ST30+ST45)	NEG	NEG	NEG	NEG	NEG	NEG	NEG	NEG
VIRULENCE : HAEMOLYSINS	hl	POS	POS	POS	POS	POS	POS	POS	POS
	hla	POS	POS	POS	POS	POS	POS	POS	POS
	hlIII (cons)	POS	POS	POS	POS	POS	POS	POS	POS
	hlIII (other than RF122)	POS	POS	POS	POS	POS	POS	POS	POS
	hlb-probe 1	POS	POS	POS	POS	AMB	POS	POS	POS

	hlb-probe 2	POS	POS	POS	POS	POS	POS	POS	POS
	hlb-probe 3	POS	POS	POS	POS	NEG	POS	POS	POS
	un-truncated hlb	NEG	NEG	NEG	NEG	NEG	POS	NEG	POS
VIRULENCE : HLB-CONV PHAGES	sak	POS	POS	POS	POS	POS	POS	POS	POS
	chp	POS	POS	POS	POS	NEG	NEG	NEG	POS
	scn	POS	POS	POS	POS	POS	POS	POS	POS
VIRULENCE : EXFOL.TOXINS	etA	NEG	NEG	NEG	NEG	NEG	NEG	NEG	NEG
	etB	NEG	NEG	NEG	NEG	NEG	NEG	NEG	NEG
	etD	NEG	NEG	NEG	NEG	NEG	NEG	NEG	NEG
VIRULENCE : EPITHEL. DIFF. INHIB	edinA	NEG	NEG	NEG	NEG	NEG	NEG	NEG	NEG
	edinB	NEG	NEG	NEG	NEG	NEG	NEG	NEG	NEG
	edinC	NEG	NEG	NEG	NEG	NEG	NEG	NEG	NEG
VIRULENCE : ACME LOCUS	ACME (total)	POS	POS	POS	NEG	NEG	NEG	NEG	NEG
	arcA-SCC	POS	POS	POS	NEG	NEG	NEG	NEG	NEG
	arcB-SCC	POS	POS	POS	NEG	NEG	NEG	NEG	NEG
	arcC-SCC	POS	POS	POS	NEG	NEG	NEG	NEG	NEG
	arcD-SCC	POS	POS	POS	NEG	NEG	NEG	NEG	NEG
VIRULENCE : PROTEASES	aur (cons)	POS	POS	POS	POS	POS	POS	POS	POS
	aur (Other than MRSA252)	POS	POS	POS	POS	POS	POS	POS	POS
	aur (MRSA252)	NEG	NEG	NEG	NEG	AMB	NEG	NEG	NEG
	splA	POS	POS	POS	NEG	NEG	NEG	NEG	NEG
	splB	POS	POS	POS	NEG	NEG	NEG	NEG	NEG
	splE	POS	POS	POS	NEG	NEG	NEG	NEG	NEG
	sspA	POS	POS	POS	POS	POS	POS	POS	POS
	sspB	POS	POS	POS	POS	POS	POS	POS	POS
	sspP (cons)	POS	POS	POS	POS	POS	POS	POS	POS
	sspP (other than ST93)	POS	POS	POS	POS	POS	POS	POS	POS
VIRULENCE : STAPHYLOCOCCAL SUPERANTIGEN/ENTEROTOXIN-LIKE GENES (SET/SSL)	setC	POS	POS	POS	POS	POS	POS	POS	POS
	set6-var1_11	N/A	N/A	N/A	POS	POS	NEG	NEG	NEG
	set6-var2_11	N/A	N/A	N/A	NEG	POS	POS	POS	POS
	set6-var1_12	N/A	N/A	N/A	AMB	POS	NEG	NEG	NEG
	set6-var2_12	N/A	N/A	N/A	NEG	POS	POS	POS	POS
	set6-var4_11	N/A	N/A	N/A	POS	NEG	NEG	NEG	NEG
	ssl01-RF122	NEG	NEG	NEG	NEG	NEG	NEG	NEG	NEG
	ssl01/set6 (COL)	POS	POS	POS	POS	NEG	NEG	NEG	NEG
	ssl01/set6 (Mu50+N315)	AMB	NEG	AMB	AMB	AMB	NEG	NEG	NEG
	ssl01/set6 (MW2+MSSA476)	NEG	NEG	NEG	NEG	POS	POS	POS	POS
	ssl01/set6 (MRSA252)	NEG	NEG	NEG	NEG	AMB	NEG	NEG	NEG
	ssl01/set6 (RF122)	N/A	N/A	N/A	N/A	NEG	NEG	N/A	N/A
	ssl01/set6 (other alleles)	NEG	NEG	NEG	NEG	NEG	NEG	NEG	NEG
	ssl02/set7	POS	POS	POS	POS	AMB	POS	POS	POS
	ssl02/set7 (MRSA252)	AMB	NEG	AMB	NEG	NEG	NEG	NEG	NEG
	ssl03/set8_probe 1	POS	POS	POS	POS	POS	POS	POS	POS
	ssl03/set8_probe 2	POS	POS	POS	POS	POS	POS	POS	POS
	ssl03/set8 (MRSA252, SAR0424)	NEG	NEG	NEG	NEG	NEG	NEG	NEG	NEG
	ssl04/set9	POS	POS	POS	POS	POS	POS	POS	POS
	ssl04/set9 (MRSA252, SAR0425)	NEG	NEG	NEG	NEG	NEG	NEG	NEG	NEG
	ssl05/set3_probe 1	POS	POS	POS	POS	POS	POS	POS	POS
	ssl05/set3 (RF122, probe-611)	AMB	AMB	NEG	AMB	POS	POS	POS	POS
	ssl05/set3_probe 2 (612)	POS	POS	POS	POS	NEG	AMB	AMB	AMB
	ssl05/set3 (MRSA252)	NEG	NEG	NEG	NEG	NEG	NEG	NEG	NEG
	ssl06/set21	NEG	POS	POS	POS	NEG	NEG	NEG	NEG
	ssl06 (NCTC8325+MW2)	POS	POS	POS	POS	NEG	NEG	NEG	NEG
	ssl07/set1	POS	POS	POS	POS	POS	POS	POS	POS
	ssl07/set1 (MRSA252)	AMB	AMB	AMB	AMB	AMB	AMB	AMB	AMB
VIRULENCE : STAPHYLOCOCCAL SUPERANTIGEN/ENTEROTOXIN-LIKE GENES (SET/SSL)	ssl07/set1 (AF188836)	AMB	NEG	NEG	AMB	NEG	NEG	NEG	AMB
	ssl08/set12_probe 1	POS	POS	POS	POS	POS	POS	POS	POS
	ssl08/set12_probe 2	POS	POS	POS	POS	POS	POS	POS	POS
	ssl09/set5_probe 1	POS	AMB	POS	POS	POS	POS	POS	POS
	ssl09/set5_probe 2	POS	POS	POS	POS	AMB	POS	POS	POS
	ssl09/set5 (MRSA252)	NEG	NEG	NEG	NEG	NEG	NEG	NEG	NEG
	ssl10/set4	POS	POS	POS	POS	POS	POS	POS	POS
	ssl10 (RF122)	POS	NEG	NEG	NEG	NEG	NEG	NEG	NEG
	ssl10/set4 (MRSA252)	NEG	NEG	NEG	NEG	NEG	AMB	NEG	AMB
	ssl11/set2 (COL)	POS	POS	POS	POS	NEG	NEG	NEG	NEG
	ssl11+set2(Mu50+N315)	NEG	NEG	NEG	NEG	NEG	NEG	NEG	NEG
	ssl11+set2(MW2+MSSA476)	NEG	NEG	NEG	NEG	NEG	NEG	NEG	NEG
	ssl11/set2 (MRSA252)	NEG	NEG	NEG	NEG	NEG	NEG	NEG	NEG
	setB3	POS	POS	POS	POS	POS	POS	POS	POS
	setB3 (MRSA252)	NEG	NEG	NEG	NEG	NEG	NEG	NEG	NEG
	setB2	POS	POS	POS	POS	POS	POS	POS	POS
	setB2 (MRSA252)	NEG	NEG	NEG	NEG	NEG	NEG	NEG	NEG

	setB1	POS	POS	POS	POS	POS	POS	POS	POS
CAPSULE- AND BIOFILM-ASSOCIATED GENES	cap 1	NEG	NEG	NEG	NEG	NEG	NEG	NEG	NEG
	capH1	NEG	NEG	NEG	NEG	NEG	NEG	NEG	NEG
	capJ1	NEG	NEG	NEG	NEG	NEG	NEG	NEG	NEG
	capK1	NEG	NEG	NEG	NEG	NEG	NEG	NEG	NEG
	cap 5	POS	POS	POS	POS	NEG	NEG	NEG	NEG
	capH5	POS	POS	POS	POS	NEG	NEG	NEG	NEG
	capJ5	POS	POS	POS	POS	NEG	NEG	NEG	NEG
	capK5	POS	POS	POS	POS	N/A	NEG	NEG	NEG
	cap 8	NEG	NEG	NEG	NEG	POS	POS	POS	POS
	capH8	NEG	NEG	NEG	NEG	POS	POS	POS	POS
	capI8	NEG	NEG	NEG	NEG	POS	POS	POS	POS
	capJ8	NEG	NEG	NEG	NEG	POS	POS	POS	POS
	capK8	NEG	NEG	NEG	NEG	POS	POS	POS	POS
	icaA	POS	POS	POS	POS	POS	POS	POS	POS
	icaC	POS	POS	POS	POS	POS	POS	POS	POS
	icaD	POS	POS	POS	POS	POS	POS	POS	POS
	bap	NEG	NEG	NEG	NEG	NEG	NEG	NEG	NEG
ADHAESION FACTORS / GENES ENCODING MICROBIAL SURFACE COMPONENTS ADHESIVE MATRIX MOLECULES (MSCRAMM GENES) RECOGNIZING	bbp	POS	POS	POS	POS	NEG	NEG	POS	POS
	bbp (cons)	POS	POS	POS	POS	NEG	NEG	POS	POS
	bbp (COL+MW2)	POS	POS	POS	POS	NEG	NEG	POS	POS
	bbp (MRSA252)	NEG	NEG	NEG	NEG	NEG	NEG	NEG	NEG
	bbp (Mu50)	NEG	NEG	NEG	NEG	NEG	NEG	NEG	NEG
	bbp (RF122)	NEG	NEG	NEG	NEG	NEG	NEG	NEG	NEG
	bbp (ST45)	NEG	NEG	NEG	NEG	NEG	NEG	AMB	AMB
	clfA	POS	POS	POS	POS	POS	POS	POS	POS
	clfA (cons)	POS	POS	POS	POS	POS	POS	POS	POS
	clfA (COL+RF122)	POS	POS	POS	POS	NEG	NEG	NEG	NEG
	clfA (MRSA252)	POS	POS	AMB	AMB	NEG	NEG	NEG	NEG
	clfA (Mu50+MW2)	POS	POS	AMB	AMB	POS	POS	POS	POS
	clfB	POS	POS	POS	POS	POS	POS	POS	POS
	clfB (cons)	POS	POS	POS	POS	POS	POS	POS	POS
	clfB (COL+Mu50)	POS	POS	POS	POS	POS	POS	POS	POS
	clfB (MW2)	NEG	NEG	NEG	NEG	NEG	NEG	NEG	NEG
	clfB (RF122)	NEG	NEG	NEG	NEG	NEG	NEG	NEG	NEG
	cna	NEG	NEG	NEG	NEG	NEG	NEG	NEG	NEG
	ebh (cons)	POS	POS	POS	POS	POS	POS	POS	POS
	ebpS	POS	POS	POS	POS	POS	POS	POS	POS
	ebpS_probe 612	POS	POS	POS	POS	POS	POS	POS	POS
	ebpS_probe 614	POS	POS	POS	POS	POS	POS	POS	POS
	ebpS (01-1111)	NEG	NEG	NEG	NEG	NEG	NEG	NEG	NEG
	ebpS (COL)	POS	POS	POS	POS	POS	POS	POS	POS
	eno	POS	POS	POS	POS	POS	POS	POS	POS
	fib	POS	POS	POS	POS	POS	POS	POS	POS
	fib (MRSA252)	NEG	NEG	NEG	NEG	NEG	NEG	NEG	NEG
	fnbA	POS	POS	POS	POS	POS	POS	POS	POS
	fnbA (cons)	POS	POS	POS	POS	POS	POS	POS	POS
	fnbA (COL)	POS	POS	POS	POS	NEG	NEG	NEG	NEG
	fnbA (MRSA252)	NEG	NEG	NEG	NEG	NEG	NEG	NEG	NEG
	fnbA (Mu50+MW2)	NEG	NEG	NEG	NEG	NEG	NEG	NEG	NEG
	fnbA (RF122)	NEG	NEG	NEG	NEG	NEG	NEG	NEG	NEG
ADHAESION FACTORS / GENES ENCODING MICROBIAL SURFACE COMPONENTS RECOGNIZING ADHESIVE MATRIX MOLECULES (MSCRAMM GENES)	fnbB	POS	POS	POS	POS	POS	POS	POS	POS
	fnbB (COL)	POS	POS	AMB	POS	NEG	NEG	NEG	NEG
	fnbB (COL+Mu50+MW2)	POS	POS	POS	AMB	POS	POS	POS	POS
	fnbB (Mu50)	POS	NEG	NEG	AMB	NEG	NEG	NEG	AMB
	fnbB (MW2)	NEG	NEG	NEG	NEG	NEG	NEG	NEG	NEG
	fnbB (ST15)	NEG	NEG	NEG	NEG	NEG	AMB	AMB	AMB
	fnbB (ST45-2)	NEG	NEG	NEG	NEG	NEG	NEG	NEG	NEG
	map	POS	POS	POS	POS	POS	POS	POS	POS
	map (COL)	POS	POS	POS	POS	NEG	NEG	NEG	POS
	map (MRSA252)	NEG	NEG	NEG	NEG	NEG	NEG	NEG	NEG
	map (Mu50+MW2)	AMB	NEG	NEG	NEG	POS	POS	POS	POS
	sasG	POS	POS	POS	POS	POS	POS	POS	POS
	sasG (COL+Mu50)	POS	POS	POS	POS	NEG	NEG	NEG	NEG
	sasG (MW2)	NEG	NEG	NEG	NEG	POS	POS	POS	POS
	sasG (OtherThan252+122	POS	POS	POS	POS	POS	POS	POS	POS
	sdrC	POS	POS	POS	POS	POS	POS	POS	POS
	sdrC (cons)	POS	POS	POS	POS	POS	POS	POS	POS
	sdrC (B1)	NEG	NEG	NEG	NEG	NEG	NEG	NEG	NEG
	sdrC (COL)	POS	POS	POS	POS	POS	POS	POS	POS
	sdrC (Mu50)	NEG	NEG	NEG	NEG	NEG	NEG	NEG	NEG
	sdrC (MW2+MRSA252+RF122)	AMB	NEG	NEG	NEG	NEG	NEG	NEG	NEG
	sdrC (OtherThan252+RF122)	POS	POS	POS	POS	POS	POS	POS	POS

	sdrD	POS	POS	POS	POS	POS	POS	POS	POS
	sdrD (cons)	POS	POS	POS	POS	POS	POS	POS	POS
	sdrD (COL+MW2)	POS	POS	POS	POS	NEG	NEG	NEG	NEG
	sdrD (Mu50)	NEG	NEG	NEG	NEG	NEG	NEG	NEG	NEG
	sdrD (other)	NEG	NEG	NEG	NEG	POS	POS	POS	POS
	vwb	POS	POS	POS	POS	POS	POS	POS	POS
	vwb (cons)	POS	POS	POS	POS	POS	POS	POS	POS
	vwb (COL+MW2)	POS	POS	POS	POS	NEG	NEG	NEG	NEG
	vwb (MRSA252)	NEG	NEG	NEG	NEG	NEG	NEG	NEG	NEG
	vwb (Mu50)	NEG	NEG	NEG	NEG	POS	POS	POS	POS
	vwb (RF122)	NEG	NEG	NEG	NEG	AMB	NEG	NEG	NEG
IMMUNOD.AG.B	isaB	POS	POS	POS	POS	POS	AMB	POS	POS
	isaB (MRSA252)	AMB	AMB	AMB	AMB	AMB	POS	AMB	AMB
DEFENSIN RESIST.	mprF (COL+MW2)	POS	POS	POS	POS	POS	POS	POS	POS
	mprF (Mu50+252)	AMB	AMB	NEG	AMB	AMB	AMB	AMB	AMB
TRANSFERRIN BINDING PROT	isdA (cons)	POS	POS	POS	POS	POS	POS	POS	POS
	isdA (MRSA252)	NEG	NEG	NEG	NEG	NEG	NEG	NEG	NEG
	isdA (Other Than MRSA252)	POS	POS	POS	POS	POS	POS	POS	POS
PUTATIVE TRANSPORTER	lmrP (OtherThanRF122)	POS	POS	POS	POS	POS	POS	POS	POS
	lmrP (OtherThanRF122)	POS	POS	POS	POS	POS	POS	POS	POS
	lmrP (RF122)	NEG	NEG	NEG	NEG	NEG	NEG	NEG	NEG
	lmrP (RF122)	NEG	NEG	NEG	NEG	NEG	NEG	NEG	NEG
TYPE I RESTRICTION-MODIFICATION SYSTEM, SINGLE SEQUENCE SPECIFICITY PROTEIN	hsdS1-RF122	NEG	NEG	NEG	NEG	NEG	NEG	NEG	NEG
	hsdS2-ST5+ST8	POS	POS	POS	POS	NEG	NEG	NEG	NEG
	hsdS2-MW2+476	NEG	NEG	NEG	NEG	POS	POS	POS	POS
	hsdS2-RF122	NEG	NEG	NEG	NEG	NEG	NEG	NEG	NEG
	hsdS2-MRSA252	NEG	NEG	NEG	NEG	NEG	NEG	NEG	NEG
	hsdS3-AllOtherThanRF122+252	POS	POS	POS	NEG	NEG	NEG	NEG	NEG
	hsdS3-ST8+ST1+RF122	POS	POS	POS	NEG	NEG	NEG	NEG	NEG
	hsdS3-Mu50+N315	NEG	NEG	NEG	NEG	NEG	NEG	NEG	NEG
	hsdS3-CC51+252	NEG	NEG	NEG	NEG	NEG	NEG	NEG	NEG
	hsdS3-MRSA252	NEG	NEG	NEG	NEG	NEG	NEG	NEG	NEG
	hsdSx-CC25	POS	POS	POS	POS	NEG	NEG	NEG	NEG
	hsdSx-CC15	NEG	NEG	NEG	NEG	NEG	NEG	NEG	AMB
	hsdSx-etd	NEG	NEG	NEG	NEG	NEG	NEG	NEG	NEG
MISCELLANEOUS GENES	Q2FXC0	POS	POS	POS	NEG	NEG	NEG	NEG	NEG
	Q2YUB3	NEG	NEG	AMB	NEG	NEG	NEG	NEG	AMB
	Q7A4X2	NEG	NEG	NEG	NEG	POS	POS	POS	POS
HYALURONATE LYASE	hysA1 (MRSA252)	NEG	NEG	NEG	NEG	NEG	NEG	NEG	NEG
	hysA1 (MRSA252+RF122) and/or hysA2 (cons)	POS	POS	POS	POS	POS	POS	POS	POS
	hysA1 (MRSA252+RF122) and/or hysA2 (COL+USA300)	POS	POS	POS	POS	POS	POS	POS	POS
	hysA2 (All Other Than MRSA252)	POS	POS	POS	POS	POS	POS	POS	POS
	hysA2 (COL+USA300+NCTC)	POS	POS	POS	POS	POS	POS	POS	POS
	hysA2 (All Other Than COL+USA300+NCTC)	NEG	NEG	NEG	NEG	NEG	NEG	NEG	NEG
	hysA2-AllOtherThan COL+USA300+NCTC	NEG	NEG	NEG	NEG	NEG	NEG	NEG	NEG
	hysA2 (MRSA252)	NEG	NEG	NEG	AMB	NEG	NEG	NEG	POS

Table S2. Results from majority voting per batch and strain for each used Raman approach

	Prediction					
Reference		MRSA	MSSA	Sensitivity (%)	Specificity (%)	Accuracy (%)
	**785 nm excitation**					
	MRSA	9	3	75	67	70.8
	MSSA	4	8			
	**UVRR**					
	MRSA	7	5	58	42	50
	MSSA	7	5			
	**532 nm excitation**					
	MRSA	7	5	58	83	70.8
	MSSA	2	10			

Table S3. Raman band assignments for the mean spectra collected with 785 nm excitation

Band /cm^{-1}	Assignment	Biomolecule	Reference
1658	Amide I (1650–1680)	Protein	(1, 2)
1523	C=C stretching vibration od staphyloxanthin (1523)	Carotinoids	(3)
1451	CH_2/CH_3 deformation of lipids (1433-1468) and proteins (1431-1481)	Protein, Lipids	(4, 5)
1337	Guanine and adenine (1337)	Nucleic acids, protein	(6)
1292	=C-H deformation (1249-1287),	Lipids	(5)
1208	Phenylalanine, Tyrosine C—C_6H_5 stretching vibration (1209)	Protein	(7, 8)
1160	C-C stretching vibration of staphyloxanthin (1159)	Carotinoids	(3)
1130	C–C stretching vibration (~1130)	Lipids	(2)
1091	PO_2 backbone symmetric stretching vibration (1100)	Nucleic acids	(9)
1031	Phenylalanine CH in plane deformation vibration (1032)	Protein	(9)
1004	Phenylalanine ring breath vibration (1004), in-plane rocking vibrations of the C-CH_3 in carotinoids (1004)	Protein	(4, 10, 11)
959	Cholesterol (~950)	Lipids	(12)
935	DNA backbone stretching vibration (929), C-C stretching vibration of proteins (929)	Nucleic acids, Proteins	(13)
896	(C - O - O) skeletal vibration of lipids (866-898)	Lipids	(5)
851	C–C stretching vibration of proline (854), Out-of-plane ring deformation vibration of tyrosine (850)	Protein	(7, 8)
782	Ring breathing modes of cytosine, uracil and thymine (780-786), O—P—O stretching vibration DNA backbone (786)	Nucleic acids	(7, 9)

Table S4. Raman band assignments for the difference spectra collected with 785 nm excitation

MSSA Band / cm^{-1}	MRSA Band / cm^{-1}	Assignment	Biomolecule	Reference
	1523	C=C stretching vibration of staphyloxanthin (1523)	Carotinoids	(3)
	1160	C-C stretching vibration od staphyloxanthin (1159)	Carotinoids	(3)
	1007	Phenylalanine ring breath vibration (1004), in-plane rocking vibrations of the C-CH_3 in carotinoids (1004)	Protein	(4, 10, 11)

Table S5. Raman band assignments for the mean spectra collected with UVRR

Band /cm^{-1}	Assignment	Biomolecule	Reference
1647	Amide I (1650–1680)	Protein	(1, 2)
1618	Tyrosine, tryptophan: in-plane C=C ring stretching vibration (1619)	Protein	(8, 10)
1575	v(ring) (C=C, C=N) of Guanine, Adenine (1578)	Nucleic acids	(2, 7)
1530	Cytosine (1529)	Nucleic acids	(14)
1483	N9C8 and C8N7 stretching vibrations along the long axis of purine bases (1485)	Nucleic acids	(12)
1416	CH_2-deformation vibration of Adenine (1420 cm^{-1})	Nucleic acids	(15)
1364	Cytosine, thymine (1369)	Nucleic acids	(10, 14)
1333	Guanine and adenine (1337)	Nucleic acids	(6)
1241	Thymine (1237)	Nucleic acids	(16)
1208	Phenylalanine, Tyrosine C—C_6H_5 stretching vibration (1209)	Protein	(7, 8)
1174	C-C and C-N stretching vibration of Cytosine and Thymine (1175), Tyrosine in-plane C-H bending vibration (1175)	Proteins, Nucleic acids	(8, 10)
1010	Phenylalanine ring breath vibration (1004)	Protein	(4, 10)
853	Out-of-plane ring deformation vibration of tyrosine (850)	Protein	(17)
829	Ring breathing vibration of Tyrosine (829)	Protein	(7, 8)
783	Ring breathing modes of cytosine, uracil and thymine (780-786), O—P—O stretching DNA backbone (786)	Nucleic acids	(7, 9)
760	Ring breathing vibration of Tryptophan (760)	Nucleic acids	(18)
726	Adenine ring streching vibration (730)	Nucleic acids	(2, 7)

Table S6. Raman band assignments for the difference spectra collected with UVRR

MSSA Band / cm^{-1}	MRSA Band / cm^{-1}	Assignment	Biomolecule	Reference
	1653	Amide I (1650–1680)	Protein	(1, 2)
	1614	Tyrosine, tryptophan: in-plane C=C stretching vibration (1619)	Protein	(8, 10)
	1554			
	1203	Phenylalanine, Tyrosine C—C_6H_5 stretching vibration (1209)	Protein	(7, 8)
	1170	C-C and C-N stretching vibration of Cytosine and Thymine (1175), Tyrosine in-plane C-H bending vibration (1175)	Proteins, Nucleic acids	(8, 10)
1581		v(ring) (C=C, C=N) of Guanine, Adenine (1578)	Nucleic acids	(2, 7)
1536		Cytosine (1529)	Nucleic acids	(14)
1486		N9C8 and C8N7 stretching vibrations along the long axis of purine bases (1485)	Nucleic acids	(12)
1320		Guanine, adenine (1316)	Nucleic acids	(16)
786		Ring breathing modes of cytosine, uracil and thymine (780-786), O—P—O str DNA backbone (786)	Nucleic acids	(7, 9)

Table S7. Raman band assignments for the mean spectra collected with 532 nm excitation

Band /cm^{-1}	Assignment	Biomolecule	Reference
2933	CH_3 stretching vibration (2909-2937)	Lipids	(5)
2888	CH_2 stretching vibration (2904-2869)	Lipids	(5)
1664	Amide I (1650–1680)	Protein	(1, 2)
1577	v(ring) (C=C, C=N) of Guanine, Adenine (1578)	Nucleic acids	(2, 7)
1448	CH_2/CH_3 deformation vibration of lipids (1433-1468) and proteins (1431-1481)	Lipids, Proteins	(4, 13, 19)
1334	Guanine and adenine (1337)	Nucleic acids, protein	(6)
1310	CH-deformation vibration (1310-1348)	Proteins	(8)
1247	Amide III random coil (1240–1265)	Protein	(8)
1127	C–C stretching vibration (~1130)	Lipids	(2)
1046	C—C and C—N stretching vibration (1057)	Proteins	(1, 8)
1004	Phenylalanine ring breathing vibration (1006)	Protein	(4)
854	C–C stretching vibration of proline (854), Out-of-plane ring deformation vibration of tyrosine (850)	Protein	(7, 8)
779	Ring breathing modes of cytosine, uracil and thymine (780-786), O—P—O stretching vibration DNA backbone (786)	Nucleic acids	(7, 9)
725	Adenine ring stretching vibration (730)	Nucleic acids	(2, 7)

Table S8. Raman band assignments for the difference spectra collected with 532 nm excitation

MSSA Band / cm^{-1}	MRSA Band / cm^{-1}	Assignment	Biomolecule	Reference
	2978	CH stretching vibration (3000-3013)	Lipids	(5)
	2945	CH_3 stretching vibration (2943-2967)	Lipids	(5)
	1673	Amide I (1650–1680)	Protein	(1, 2)
	1607	Tyrosine, tryptophan: in-plane C=C ring stretching vibration (1619)	Protein	(8, 10)
	1487	N9C8 and C8N7 stretching vibrations along the long axis of purine bases (1485)	Nucleic acids	(12)
	1418	CH deformation vibration of nucleic acids, CH_2 deformation vibration of proteins and lipids (1420)	Nucleic acids, Proteins, Lipids	(6)
	1355	CH deformation vibration of proteins (1341), Guanine and Adenine (1337)	Proteins, nucleic acids	(5, 9)
	1244	Amide III random coil (1240–1265)	Protein	(8)
	1004	Phenylalanine ring breath vibration (1004)	Protein	(4, 10)
2891		CH_2 stretching vibration (2904-2869)	Lipids	(5)
2849		CH_3 stretching vibration (2943-2967)	Lipids	(5)
1523		Cytosine (1529)	Nucleic acids	(14)
1301		CH_2 twisting vibration (1294-1306)	Lipids	(5)
1160		C-C stretching vibration of fatty acids (1166-1179)	Lipids	(5)
1127		C–C stretching vibration (~1130)	Lipids	(2)
1046		C—C and C—N stretching vibration (1057)	Proteins	(1, 8)
779		Ring breathing modes of cytosine, uracil and thymine (780-786), O—P—O stretching vibration DNA backbone (786)	Nucleic acids	(7, 9)

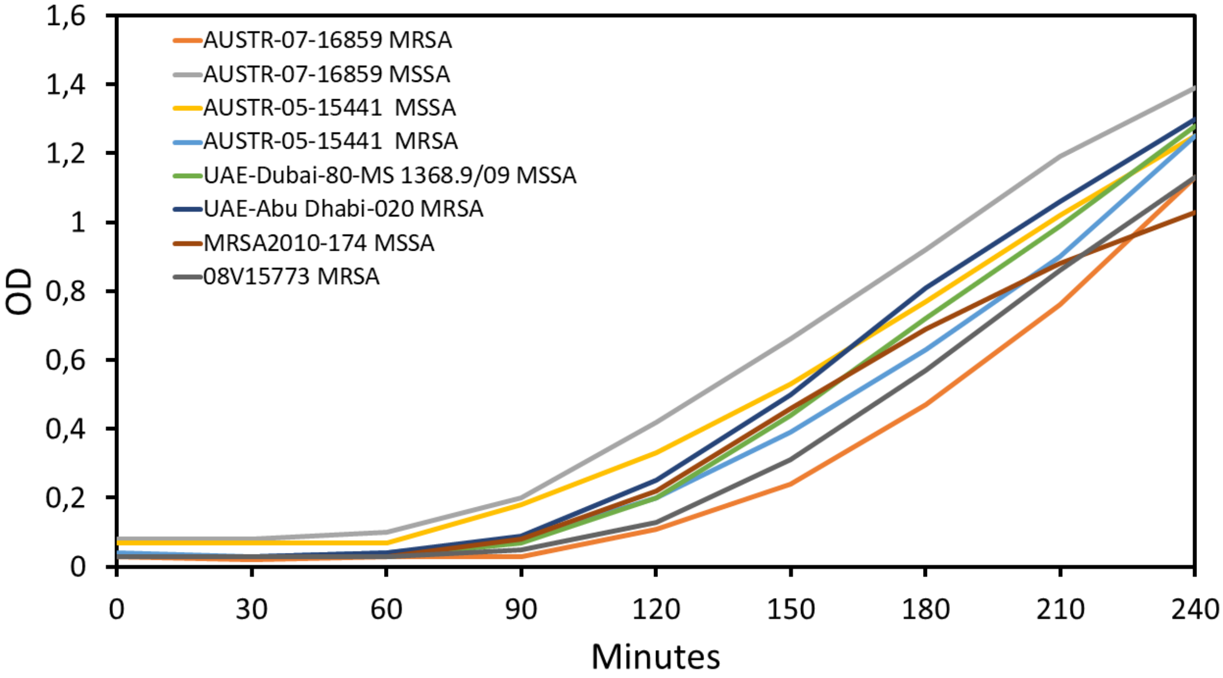

Figure S1. Grow curves of used bacterial strains

Table S9. Number of measured spectra.

	Total spectra measured	Spectra per strain	Spectra per batch	Total spectra analyzed
**785 nm excitation**	500	~60	~20	498
**UVRR**	600	75	25	598
**532 nm excitation**	1440	60	180	1276

Table S10. Classification results before majority voting.

		Prediction				
		MRSA	MSSA	Sensitivity (%)	Specificity (%)	Accuracy (%)
Reference	**785 nm excitation**					
	MRSA	161	93	63.4	65.6	64.5
	MSSA	84	160			
	**UVRR**					
	MRSA	185	113	62.5	65	63.7
	MSSA	104	196			
	**532 nm excitation**					
	MRSA	352	292	70.9	54.7	62.8
	MSSA	184	448			

Table S11. Percentage of spectra that were correctly identified for each strain of all used Raman spectroscopy approaches.

		Correctly identified strains (%)		
		**785 nm excitation**	**UVRR**	**532 nm excitation**
**AUSTR-07-16859**	MRSA	39.73	57.53	52.53
**AUSTR-07-16859**	MSSA	53.33	37.33	69.63
**08V15773**	MRSA	58.33	45.33	62.28
**MRSA2010-174**	MSSA	56.67	73.33	60.63
**AUSTR-05-15441**	MRSA	90.00	64.00	69.83
**AUSTR-05-15441**	MSSA	90.63	69.33	52.32
**UAE-Abu Dhabi-020**	MRSA	70.49	37.33	64.83
**UAE-Dubai-80-MS 1368.9/09**	MSSA	60.00	62.67	42.41

References

1. Uzunbajakava N, Lenferink A, Kraan Y, Volokhina E, Vrensen G, Greve J, Otto C. 2003. Nonresonant Confocal Raman Imaging of DNA and Protein Distribution in Apoptotic Cells. Biophysical Journal 84:3968-3981.
2. Maquelin K, Kirschner C, Choo-Smith LP, van den Braak N, Endtz HP, Naumann D, Puppels GJ. 2002. Identification of medically relevant microorganisms by vibrational spectroscopy. Journal of Microbiological Methods 51:255-271.
3. Ayala OD, Wakeman CA, Pence IJ, Gaddy JA, Slaughter JC, Skaar EP, Mahadevan-Jansen A. 2018. Drug-Resistant Staphylococcus aureus Strains Reveal Distinct Biochemical Features with Raman Microspectroscopy. ACS Infectious Diseases 4:1197-1210.
4. Töpfer N, Müller MM, Dahms M, Ramoji A, Popp J, Slevogt H, Neugebauer U. 2019. Raman spectroscopy reveals LPS-induced changes of biomolecular composition in monocytic THP-1 cells in a label-free manner. Integr Biol (Camb) doi:10.1093/intbio/zyz009.
5. Czamara K, Majzner K, Pacia MZ, Kochan K, Kaczor A, Baranska M. 2015. Raman spectroscopy of lipids: a review. Journal of Raman Spectroscopy 46:4-20.
6. De Gelder J, De Gussem K, Vandenabeele P, Moens L. 2007. Reference database of Raman spectra of biological molecules. Journal of Raman Spectroscopy 38:1133-1147.
7. Huang WE, Li M, Jarvis RM, Goodacre R, Banwart SA. 2010. Shining light on the microbial world the application of Raman microspectroscopy. Adv Appl Microbiol 70:153-86.
8. Azemtsop Matanfack G, Pistiki A, Rösch P, Popp J. 2021. Raman Stable Isotope Probing of Bacteria in Visible and Deep UV-Ranges. Life 11.
9. Neugebauer U, Clement JH, Bocklitz T, Krafft C, Popp J. 2010. Identification and differentiation of single cells from peripheral blood by Raman spectroscopic imaging. J Biophotonics 3:579-87.
10. Harz M, Krause M, Bartels T, Cramer K, Rösch P, Popp J. 2008. Minimal Invasive Gender Determination of Birds by Means of UV-Resonance Raman Spectroscopy. Analytical Chemistry 80:1080-1086.
11. Kumar V, Kampe B, Rösch P, Popp J. 2015. Classification and identification of pigmented cocci bacteria relevant to the soil environment via Raman spectroscopy. Environ Sci Pollut Res Int 22:19317-25.

12. Germond A, Ichimura T, Horinouchi T, Fujita H, Furusawa C, Watanabe TM. 2018. Raman spectral signature reflects transcriptomic features of antibiotic resistance in Escherichia coli. Communications Biology 1:85.
13. Managò S, Zito G, De Luca AC. 2018. [INVITED] Raman microscopy based sensing of leukemia cells: A review. Optics & Laser Technology 108:7-16.
14. Walter A, Reinicke M, Bocklitz T, Schumacher W, Rösch P, Kothe E, Popp J. 2011. Raman spectroscopic detection of physiology changes in plasmid-bearing Escherichia coli with and without antibiotic treatment. Anal Bioanal Chem 400:2763-73.
15. Notingher I, Hench LL. 2006. Raman microspectroscopy: a noninvasive tool for studies of individual living cells in vitro. Expert Review of Medical Devices 3:215-234.
16. Silge A, Heinke R, Bocklitz T, Wiegand C, Hipler U-C, Rösch P, Popp J. 2018. The application of UV resonance Raman spectroscopy for the differentiation of clinically relevant Candida species. Analytical and Bioanalytical Chemistry 410:5839-5847.
17. Harz M, Claus RA, Bockmeyer CL, Baum M, Rösch P, Kentouche K, Deigner HP, Popp J. 2006. UV-resonance Raman spectroscopic study of human plasma of healthy donors and patients with thrombotic microangiopathy. Biopolymers 82:317-24.
18. Azemtsop Matanfack G, Taubert M, Guo S, Bocklitz T, Küsel K, Rösch P, Popp J. 2021. Monitoring Deuterium Uptake in Single Bacterial Cells via Two-Dimensional Raman Correlation Spectroscopy. Analytical Chemistry 93:7714-7723.
19. Benevides JM, Overman SA, Thomas Jr GJ. 2005. Raman, polarized Raman and ultraviolet resonance Raman spectroscopy of nucleic acids and their complexes. Journal of Raman Spectroscopy 36:279-299.
